# Therapeutic efficacy of pyronaridine-artesunate (Pyramax^®^) against uncomplicated *Plasmodium falciparum* infection at Hamusit Health Centre, Northwest Ethiopia

**DOI:** 10.1186/s12936-023-04618-y

**Published:** 2023-06-17

**Authors:** Mihreteab Alebachew, Woyneshet Gelaye, Megbaru Alemu Abate, Heven Sime, Henok Hailgiorgis, Bokretsion Gidey, Mebrahtom Haile, Gudissa Assefa, Worku Bekele, Habtamu Belay, Jonathan B. Parr, Geremew Tasew, Hussein Mohammed, Ashenafi Assefa

**Affiliations:** 1 Department of Medical Laboratory Sciences, College of Medicine and Health Sciences, Wollo University, P.O Box 1145, Dessie, Ethiopia; 2 Department of Medical Laboratory Sciences, College of Medicine and Health Sciences, Bahir Dar University, Bahir Dar, Ethiopia; 3 Malaria and Neglected Tropical Diseases Research Team, Bacterial, Parasitic and Zoonotic Disease Research Directorate, Ethiopian Public Health Institute, Addis Ababa, Ethiopia; 4 Ethiopian Federal Ministry of Health, Addis Ababa, Ethiopia; 5 World Health Organization, Addis Ababa, Ethiopia; 6 Department of Medical Laboratory Sciences, College of Medicine and Health Sciences, Wolkite University, Wolkite, Ethiopia; 7 Institute for Global Health and Infectious Disease, University of North Carolina at Chapel Hill, Chapel Hill, NC USA; 8 The Unversity of Queensland, School of Public Health, Brisbane, Australia

**Keywords:** Efficacy, Ethiopia, Hamusit, P. falciparum, Pyronaridine-artesunate, Malaria

## Abstract

**Background:**

Early case detection and prompt treatment are important malaria control and elimination strategies. However, the emergence and rapid spread of drug-resistant strains present a major challenge. This study reports the first therapeutic efficacy profile of pyronaridine-artesunate against uncomplicated *Plasmodium falciparum* in Northwest Ethiopia.

**Methods:**

This single-arm prospective study with 42-day follow-up period was conducted from March to May 2021 at Hamusit Health Centre using the World Health Organization (WHO) therapeutic efficacy study protocol. A total of 90 adults ages 18 and older with uncomplicated falciparum malaria consented and were enrolled in the study. A standard single-dose regimen of pyronaridine-artesunate was administered daily for 3 days, and clinical and parasitological outcomes were assessed over 42 days of follow-up. Thick and thin blood films were prepared from capillary blood and examined using light microscopy. Haemoglobin was measured and dried blood spots were collected on day 0 and on the day of failure.

**Results:**

Out of 90 patients, 86/90 (95.6%) completed the 42-day follow-up study period. The overall PCR-corrected cure rate (adequate clinical and parasitological response) was very high at 86/87 (98.9%) (95% CI: 92.2–99.8%) with no serious adverse events. The parasite clearance rate was high with fast resolution of clinical symptoms; 86/90 (95.6%) and 100% of the study participants cleared parasitaemia and fever on day 3, respectively.

**Conclusion:**

Pyronaridine-artesunate was highly efficacious and safe against uncomplicated *P. falciparum* in this study population.

**Supplementary Information:**

The online version contains supplementary material available at 10.1186/s12936-023-04618-y.

## Background

Malaria continues to be an important public health problem. It is one of the major tropical diseases adversely affecting the population health and economic growth of many developing countries, particularly in sub-Saharan Africa [1]. The World Health Organization (WHO) African Region reported an estimated 234 million cases and 593,000 deaths in 2021 [1]. Ethiopia is one of the African countries where both *Plasmodium falciparum* and *Plasmodium vivax* malaria parasites co-exist. Malaria affects more than 60% of the population and is endemic across 75% of the country’s land mass. Malaria in Ethiopia is seasonal, unstable, and cases generally occur in areas with altitudes below 2000 m above sea level [2].

Artemisinin-based combination therapy (ACT) for the management of uncomplicated *P. falciparum* and *P. vivax* malaria cases is recommended worldwide. ACT reduces both malaria-related morbidity and mortality and also hampers transmission by acting on gametocytes and reducing the likelihood that drug resistance develops [3]. Pyronaridine-artesunate employs the combination of a potent, short-acting artemisinin and long-acting partner drug, and is important to ensure effective malaria case management [4]. Resistance to artemisinin was first reported along the Thai-Cambodian border and now threatens malaria control efforts in Africa [5].

Several clinical and molecular studies show that artemisinin resistance is widespread in Cambodia, Thailand, Myanmar, and Vietnam, where delayed clearance and the WHO validated partial artemisinin-resistance Kelch protein (K13) mutations are common [6]. Emerging reports of artemisinin resistance in Rwanda, Uganda, and Eritrea raise concern about the long-term viability of existing ACT regimens [7].

The development of alternative therapies and artemisinin derivatives is essential to overcome the spread of drug-resistant malaria [8]. Pyronaridine (a hydroxy anilino-benzonaphthyridine derivative) is a synthetic anti-malarial drug that has been in use for the treatment of chloroquine-resistant malaria in China since 1970. The combination of pyronaridine with other anti-malarial agents, including artesunate, has an additive effect [9, 10]. Artesunate is another artemisinin-derivative anti-malarial mainly used as intravenous treatment for children or adults with severe malaria. It has rapid action, has largely escaped clinical resistance, and is more water soluble compared to other artemisinin derivatives [7, 8]. Pyronaridine-artesunate (Pyramax^®^) is the recent artemisinin-based combination for which the European Medicines Agency (EMA) has adopted a positive scientific opinion, and it is included in the WHO list of prequalified medicines for malaria [11, 12]. Large-scale clinical studies in Africa and Asia reported high efficacy and safety for pyronaridine artesunate against uncomplicated falciparum and vivax malaria [13, 14]. This study reports the first safety and efficacy profile of pyronaridine-artesunate in Ethiopia. The study reports the 42 days follow up treatment outcome of adult out patients attending Hamusit Health Centre, reporting uncomplicated *P. falciparum* malaria.

## Methods

### Study design, period and area

A single-arm, invivo, prospective therapeutic efficacy study of pyronaridine-artesunate treatment for uncomplicated falciparum malaria was conducted, and clinical, parasitological, and haematological assessments were performed. The study was conducted from March to May 2021 in Hamusit Health Centre, Dera Woreda, South Gonder, Northwest Ethiopia (11° 43′ 0″ North and 37° 38′ 0″ East). The study area is located 2077 m above sea level, and receives 1300 mm of annual rainfall on average, with a 26 °C mean annual temperature. The area is characterized by unstable seasonal malaria that peaks following the major rainy season (June to September). The health centre serves about 54,940 people, and encompasses eight primary health units (health posts).

### Study population

The study participants from laboratory-confirmed, *P. falciparum* mono infected patients who attended the outpatient department (OPD) of the Hamusit Health Centre and fulfilled the study inclusion criteria were recruited. Standardized WHO anti-malarial drug efficacy surveillance inclusion and exclusion criteria were utilized [8]. Included patients had an asexual parasitaemia level above 500 parasites/µl, had axillary temperature above 37.5 °C or a history of fever in the past 24 h, could comply with the study protocol, and signed an informed consent. One difference to the WHO protocol was limitation of this study to adults 18 years and older by the institutional ethics committee, as the study drug is new to Ethiopia. Patients having one or more of the following were excluded from the study: having general danger signs or signs of severe malaria, haemoglobin < 8 g/dl, severe malnutrition, other non-malaria febrile condition, known chronic or severe diseases; regular medication; a history of hypersensitivity reactions or contraindications to medications and pregnancy or breastfeeding.

### Sample size determination

The sample size was determined according to the 2009 WHO protocol, using the single population proportion formula and calculated assuming a 5% margin of error, a 95% confidence interval CI 5% treatment failure and 20% adjustment for loss to follow up and withdrawal compensation. Accordingly, 88 study participants were required.

### Baseline evaluation

Physical and clinical examinations were assessed by a clinical study team, with particular attention to any danger signs or symptoms associated with severe malaria. Clinical history and demographic data were collected, axillary temperature and body weight were measured. Patients who met the study inclusion criteria at this stage were assigned a patient identification number and referred for further clinical, laboratory investigation, sample collection and enrolment.

### Clinical evaluations

A standard physical examination, body weight, axillary temperature, and clinical conditions were performed at baseline (day 0 before dosing) and on days 1, 2, 3, 7, 14, 21, 28, 35, and 42. A complete medical history, including prior and concomitant medications, demographic information, and contact details, were recorded at baseline.

### Treatment, dosing, and follow up

Drug dosage was determined according to the revised WHO protocol. Accordingly, enrolled patients were treated with the standard three-dose regimen of 180 mg/60 mg film-coated tablet pyronaridine tetra phosphate/artesunate, given once daily for 3 consecutive days. All doses of the medication were administered in the health centre under the direct supervision of the study clinical team. The doses on day 0, day 1, and day 2 were administered consecutively in 24 h interval.

The patients were observed for 30 min after drug administration. If vomiting occurred before 30 min, the dose was repeated and observed for an additional 30 min. A patient vomiting more than once was withdrawn from the study and was referred immediately to the OPD for rescue treatment with intramuscular or intravenous quinine.

On day 0 (enrollment day), each patient who was successfully treated with the first dose was given an appointment card with their name, Patient Identification Number (PIN), and next scheduled visit date written on the front of it. Then patients were asked and advised to come back for treatment the following 2 days and in a total of 42 follow-up days according to scheduled visits on day 1, day 2, day 3, day 7, day 14, day 21, day 28, day 35, and day 42.

### Laboratory procedures

#### Microscopic investigation

A commonly used microscopy-based malaria diagnosis approach was followed. Detection, identification, and quantification of parasites was made from same slide. In brief: two thick and thin blood smears were prepared as per the study protocol: the first was 10% Giemsa stained for 10–15 min for rapid initial microscopy screening, and the second one was stained slowly with 3% Giemsa for 45–60 min and examined for a definitive parasite count.

Slides were examined using light microscopy and parasite density was calculated according to the WHO protocol (8). Asexual parasite density was estimated from thick blood smears by counting the number of asexual parasites against 200 WBC or against 500 WBC (if the count was below 10 parasites per 200 WBC) assuming an average WBC count of 8000/l of blood.

$${\text{Parasite density}}\,(\mu {\text{l}}) = \,\,\frac{{{\text{Number of parasite count}}}}{{{\text{Number of WBC counted}}}}\, \times \,8000$$

### Measurement of blood haemoglobin level

Haemoglobin levels were measured from a finger-prick blood sample. Finger-prick blood samples were collected on day 0, day 14, day 28 and day 42 using HemoCue microcuvets and were measured with a portable spectrophotometer (HemoCue, Ängelhom, Sweden). Anaemia was defined according to the WHO classification to mild, moderate and severe (mild: Hb = 10.0–11.9 and 11.0–12.9 g/dl for women and men, respectively; moderate: Hb = 7.0–9.9 g/dl and 8–10.9 g/dl for women and adult men, respectively; Hb 5.0 g/dl was considered severe anaemia and an exclusion criteria) [15].

### Genotyping

Dried blood spots (DBS) were obtained for all patients on Day 0 and on days of treatment failure for polymerase chain reaction (PCR) differentiation of recrudescence (same parasite strain) from a new infection (acquired new infection by a new parasite strain). Agarose gel-based conventional PCR analysis was used on paired DBS (Day 0 and day of treatment failure), targeting commonly used *merozoite surface protein 2 (msp2)* and *glutamate-rich protein (glurp)* gene targets selected based on their genetic diversity. PCR genotyping was made by amplifying both gene targets in the paired samples, separating them in 3% agarose gel and visualize the migration difference under a UV light. PCR correction was made at the Ethiopian Public Health Institute (EPHI) and confirmed at the University of North Carolina, Chapel Hill, according to WHO recommendations [8, 16].

### Study endpoints

The WHO treatment outcome definition was used; valid study endpoints included treatment failure during the study period (early treatment failure [ETF], late clinical failure [LCF], and late parasitological failure [LPF]), completion of the follow-up period without treatment failure (adequate clinical and parasitological response [ACPR]), and loss to follow-up (LFU).

### Safety and quality assessment

Safety and adverse events were assessed by recording the nature and incidence of any events following treatment. Adverse events were classified and reported to the Ethiopian Public Health Institute, as required. Data was collected by trained senior clinicians in the study health centre and standard operating procedures were followed for each laboratory activity. The quality of the reagent and equipment was maintained as per the standard operating procedures.

### Statistical analysis

Data were double-entered into the WHO Excel sheet, which is designed for therapeutic efficacy study data analysis. Discrepancies were resolved by referring to the original paper documents. SPSS (version 20) software was used to calculate descriptive statistics (mean, median, standard deviation, and range). Independent sample t-test was used to compare baseline temperature, parasitaemia, and median blood Hb level at day 0, day 14, day 28 and day 42 between patients with parasitaemia ≥ 10,000 and < 10,000/µl blood. Paired sample t-test was used to compare the median Hb level between day 0 and day 14, day 0 and day 28, day 14 and day 28, day 0 and day 42, day 14 and day 42, day 28 and day 42. All comparisons were performed at 95% CI and a significance level of 0.05.

Kaplan Meier (K-M) survival analysis and per protocol (PP) analysis were used for the estimation of primary outcomes; PP analysis method was used to analyse secondary outcomes.

### Ethical considerations

The study protocol was approved by the Ethiopian Public Health Institute (EPHI) and by the Institutional Review Boards (IRBs) of Bahir Dar University. Permission was obtained from the Amhara regional health office and Hamusit health centre. In addition, laboratory work was reviewed and determined as non-human subjects research by the Office of Human Research Ethics at the University of North Carolina at Chapel Hill. Written consent was obtained from study participants.

## Results

### Characteristics of the study participants

A total of 4372 malaria suspected symptomatic out patients visiting Hamusit Health Centre were screened for malaria during the study period. Of these, 427 (9.8%) were malaria microscopy slide positive; 345 (81%) were *P. falciparum*, 82 (19%) were *P. vivax*. Most of those patients were aged less than 18 years and consequently excluded from the study. A total of 109 patients were eligible to be included in the study. Five patients refused to give consent, seven were with parasitaemia below 1000/µL, and seven were lactating mothers; were consequently excluded.

Ninety patients were qualified and enrolled. At baseline, 78/90 (86.7%) patients were febrile and 19/90 (21.1%) patients were anaemic (5/90 (5.5%) moderate and 14/90 (15.6%) mild anaemia). Overall, 17/19 (89.5%) of the 19 anaemic patients were males (Table 1).

**Table 1:** Base line characteristics of the study participants at Hamusit health centre, northwest Ethiopia, 2021

Variables		Result
Median age (range) in years		27.5 (18–76)
Sex		
Male		68/90 (75.6)
Female		22/90 (24.4)
Median body temperature		38.0 ± 0.94
Average median body weight		52.0 (48–56.1)
Haemoglobin level		14.2 ± 2.03
Median parasitaemia (range)		6000 (1280–22,200)
Bed net availability at home		
Yes		28/90 (31.1%)
No		62/90 (68.9%)
Previous malaria attack		
Yes		60/90 (66.7%)
No		30/90 (33.3%)

**Fig. 1:**
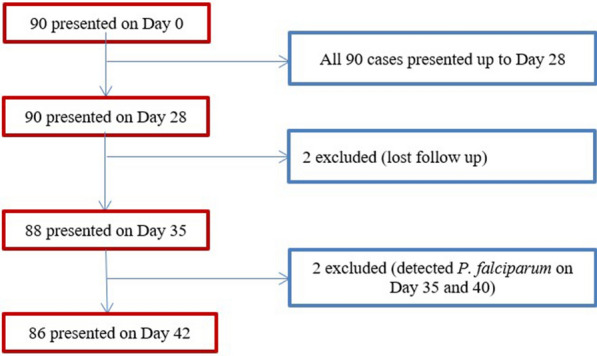
Study participants’ flow chart

Among the 90 participants, 4 exclusions were recorded on different follow-up days. Two were excluded on day 28 due to LFU, two had *P. falciparum* infection on day 35 and on day 40. 1). All 90 enrolled patients were presented up to the 28th follow up day, 88 cases were presented on day 35, and 86 cases were presented on day 42 (Fig. 1).

Sex. Bed net availability at home and Previous malaria attack were described as number (n) and percentile (%), Median body temperature was measured by °C. Haemoglobin level was measured as gram per decilitre (g/dl) and median body temperature and haemoglobin level were described as Mean ± Standard deviation (M ± SD) Average median body weight was described as kilogram (kg) and interquartile range (IQR), median parasitaemia was quantified per µl.

### Cure rate

The PCR-corrected cure rate of pyronaridine-artesunate was 86/87 (98.9%) based on the PP analysis method with one LPF (parasitaemia 4520/µl on Day 35) and no ETF or LCF Additional file: 1 (Table 2). The overall PCR-corrected cure rate by K-M survival estimate was 86/87 98.9% (95% CI 92.2–99.8%) (Table 3). PCR correction classified one of the two patients as recrudescence (day 35) and the other patient as recurrence (day 40) in this study.

**Table 2:** Summary of treatment 42 days treatment outcomes based on PP analysis among patients who treated Pyramax at Hamusit Health Centre, Northwest Ethiopia, 2021

Treatment outcomes	Frequency (%)
ETF	0 (0)
LCF	0 (0)
LPF	2/90 (2.3%)
TTF	2/90 (2.3%)
ACPR	86/87 (98.9%)
LFU	2/90 (2.2%)
Withdrawal	0 (0)

*ACPR* Adequate clinical and parasitological response, *ETF* Early treatment failure, *LCF* Late clinical failure, *LFU* Lost follow up, *LPF* Late parasitological failure, *N* number, *TTF* Total treatment failure

**Table 3:** Summary of PCR-corrected cure rate based on K-M survival analysis at Hamusit Health Centre, Northwest Ethiopia, 2021

Follow up days	N	Censored	Failure	Survived	K-M success	K-M failure
0	90	0	0	90	1	0
1	90	0	0	90	1	0
2	90	0	0	90	1	0
3	90	0	0	90	1	0
7	90	0	0	90	1	0
14	90	0	0	90	1	0
21	90	0	0	90	1	0
28	90	2	0	90	1	0
35	88	0	1	87	0.988636	0.011364
42	87	0	1	86	0.988506	0.011494

### Clearance of parasitaemia following treatment

**Fig. 2:**
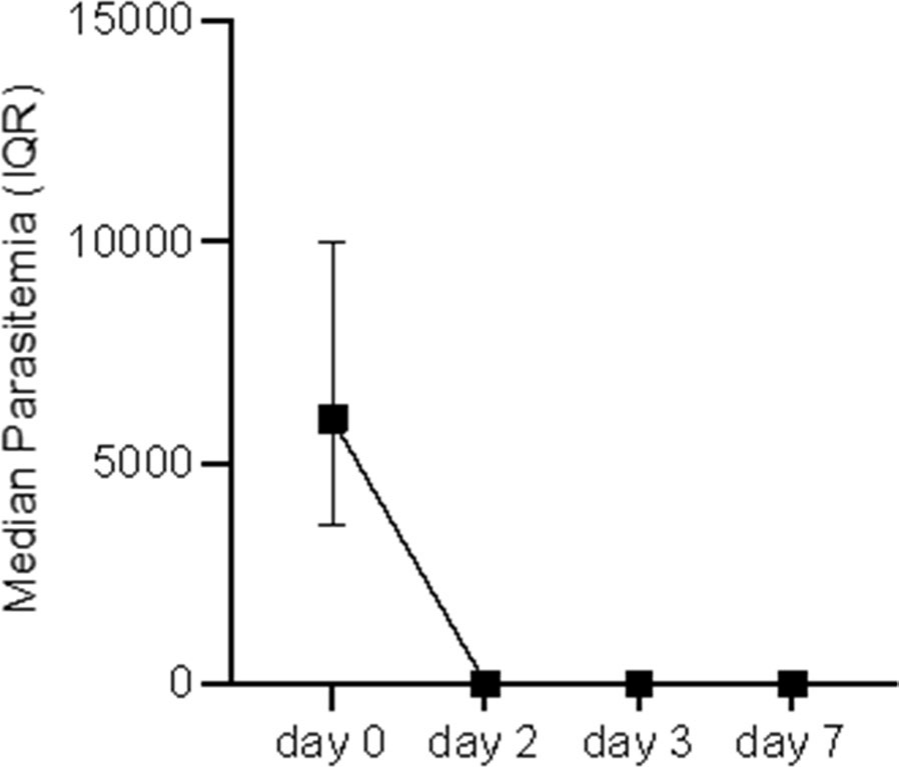
Median parasitaemia on the first three follow-up days of Pyronaridine-artesunate treatment at Hamusit Health Centre, Northwest Ethiopia, 2021

Parasite clearance rate was high, 78/90 (86.7%) of the patients cleared parasitaemia on Day 2, 86/90 (95.6%) on Day 3 and 100% on Day 7. The median parasitaemia declined from 6000 on Day 0 (baseline) to 127 parasites/µl on Day 2, 90 on Day 3 and 0 on Day 7 (Fig. 2).

### Progress of clinical signs and symptoms

**Fig. 3:**
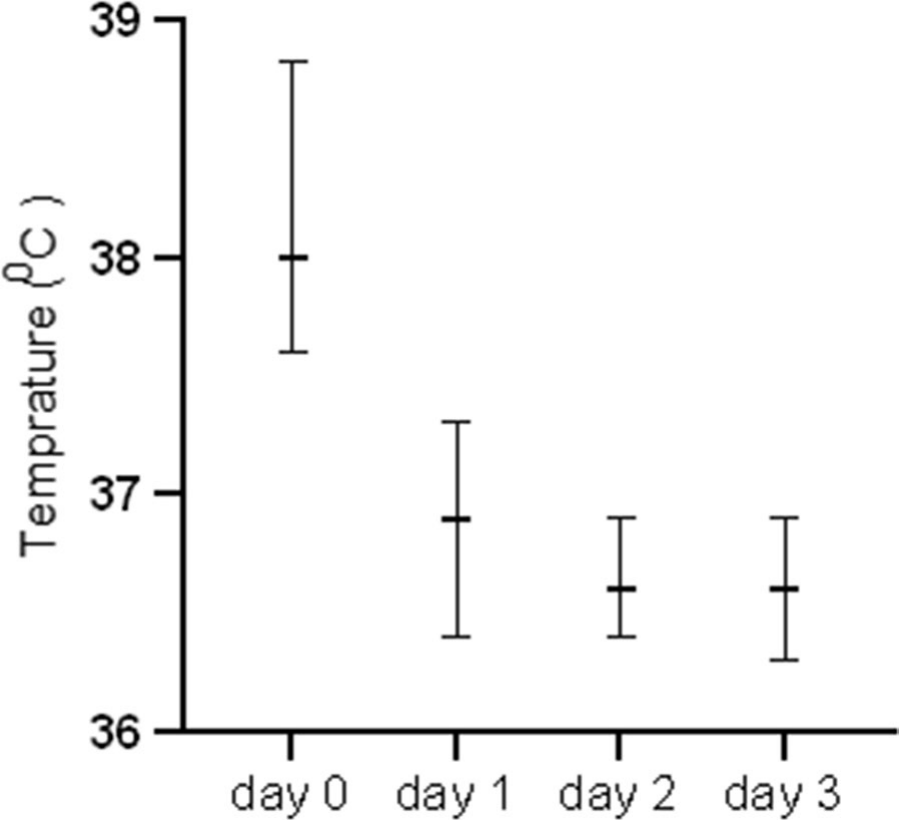
Median body temperature on the first 3 follow-up days of Pyronaridine–artesunate treatment at Hamusit Health Centre, Northwest Ethiopia, 2021

At baseline, 78/90 (86.7%) had a fever during enrollment. All the participants had self-reported fever within 24 h before enrollment. The median body temperature declined from 38.00 C on Day 0 to 36.90 C on Day 1, 36.60 C on Day 2 and to 36.60 C on Day 3 (Fig. 3), with 72/90 (80%) of the participants clearing fever on Day 1, and 100% on Day 3.

At baseline, a number of signs and symptoms were reported. Headaches were reported by all patients. The following signs and symptoms were reported by the participants: anorexia 26/90 (29%), nausea 19/90 (21.1%), dizziness 17/90 (19%), abdominal pain 13/90 (14.4%), diarrhea 12/90 (13.3%), vomiting 15/90 (16%), and cough 2/90 (2.2%). During follow-up, some of these events persisted among some participants and new ones also emerged in total of 62/90 (68.9%) participants (Table 4.). Most of the AEs disappeared with the clearance of parasitaemia except headache, cough, abdominal pain, and mouth ulcer.

**Table 4:** Probable drug-related AEs observed during the study

Adverse events	Follow-up days and number (frequency) of adverse events (N = 90)										
	Day-0	Day-1	Day-2	Day-3	Day-7	Day-14	Day-21	Day-28	Day-35	Day-42	Total n (%)
Headache	5 (5.5)	3 (3.3)	3 (3.3)	–	–	–	–	–	–	–	11 (12.2)
Anorexia	2 (2.2)	4 (4.4)	3 (3.3)	–	–	–	–	–	–	–	9 (10.0)
Mouth ulcer	2 (2.2)	4 (4.4)	2 (2.2)	1(1.1)	–	–	–	–	–	–	9 (10.0)
Vomiting	2 (2.2)	1 (1.1)	–	–	–	–	–	–	–	–	3 (3.3)
Abdominal pain	–	3 (3.3)	3 (3.3)	2 (2.2)	–	–	–	–	–	–	8 (8.9)
Diarrhea	–	1 (1.1)	2 (2.2)	2 (2.2)	–	–	–	–	–	–	5 (5.6)
Cough	2 (2.2)	2 (2.2)	2 (2.2)	2 (2.2)	1 (1.1)	–	–	–	–	–	9 (10.0)
Dizziness	3 (3.3)	2 (2.2)	2 (2.2)	1 (1.1)	–	–	–	–	–	–	8 (8.9)
Total n (%)	16 (17.6)	20 (22.0)	17 (18.7)	8 (8.8)	1 (1.1)	–	–	–	–	–	62 (68.9)

### Prevalence of anaemia

The proportion of moderately and mildly anaemic patients was 17/90 (19%) at baseline and had risen to 40/90 (44%), 27/90 (30%) and 22/86 (25%) on Days 14, 28, and 42, respectively (Table 5.). Comparison of Day 0, Day 14, Day 28 and Day 42 median Hb level between patients with parasitaemia ≥ 10,000 and < 10,000/µl showed no significant difference 14.4 ± 1.96, 14.1 ± 2.05, p = 0.452; 12.5 ± 1.49, 12.8 ± 1.87, p = 0.534; 13.6 ± 1.68, 13.5 ± 1.47, p = 0.436, 13.9 ± 2.03, 13.3 ± 1.68, p = 0.201 for ≥ 10,000 and < 10,000/µl; and for Day 0, Day 14 Day 28 and Day 42, respectively.

**Table 5:** Median haemoglobin concentration (g/dl) of study population during follow-up days at Hamusit Health Centre, Northwest Ethiopia, 2021

Variables			Follow up days			
			Day 0	Day 14	Day 28	Day 42
Median Hb±SD (g/dl)			14.2±2.03	12.7±1.75	13.4±1.52	13.5±1.79
Anaemia status						
Mild n (%)		Male	14 (21.2)	27 (40.9)	20 (30.3)	18 (27.3)
		Female	0 (0)	3 (15)	4 (20)	2 (10)
		Total	14 (21.2)	30 (55.9)	24 (50.3)	20 (37.3)
Moderate (%)		Male	3 (4.5)	12 (18.2)	6 (9.1)	5 (7.5)
		Female	2 (10)	2 (10)	0 (0)	0 (0)
		Total	5 (15.5)	14 (18.2)	6 (9.1)	5 (7.5)
Total (%)			19 (22.1)	44 (51.2)	30 (34.9)	25 (29.1)

## Discussion

In this first study in Ethiopia, pyronaridine-artesunate showed high therapeutic efficacy, with a 86/87 (98.9%) PCR-corrected cure rate. This estimate is in line with studies done in five African countries (98.6%), Koh Gnek (98.3%), central and southern Vietnam (96.1%), and Veun Sai of eastern Cambodia (96.7%) [17–20]. A study on the efficacy of pyronaridine–artesunate and artemether–lumefantrine showed 98.9% efficacy for pyronaridine-artesunate and 96.4% efficacy, respectively, with pyronaridine–artesunate not inferior to artemether–lumefantrine [20]. However, the cure rate of the pyronaridine–artesunate in the present study was higher than a similar study in western Cambodia (87.9%) [21]. It is possible that efficacy is lower in Cambodia because parasites are resistant to pyronaridine-artesunate or recrudescence was overestimated by using PCR methods on a clonal parasite population. Reinfection with the clonal parasite population would not be easily distinguishable from recrudescence [21].

The absence of ETF and low parasitological failure (only single LPF) in this study indicates the high therapeutic efficacy of pyronaridine-artesunate in the study setting. Studies from Vietnam and western Cambodia reported six and fifteen late treatment failures after 21 follow-up days of pyronaridine–artesunate treatment [22–24]. This might be due to insufficient drug levels or parasite resistance to this drug. Artemisinin has a short half-life of 1–3 h, resulting in a period below the low minimum inhibitory concentration (MIC) required to kill all parasites [22]. Moreover, the asexual blood stage *P. falciparum* parasites may become temporarily dormant and survive the therapeutic concentration of artemisinin derivatives [25]. However, the PCR-confirmed recrudescence reported in this study warrants follow-up and advanced molecular screening for resistance markers.

In the current study, 86/90 (95.6%) of patients cleared parasitaemia by day 3 [median parasitaemia 0.0, Interquartile range (IQR) (0.0–120.0)/ µl], which is consistent with a study done in Myanmar [19]. Other previous studies reported lower rates of parasite clearance: 74% in Vietnam, 56.4% in Pailin, and 86.7% in Pursat on the third day of drug administration [22, 25]. The parasite clearance rate can be affected by drug blood concentration profiles, host–defense mechanisms, the initial parasitaemia, concomitant infection, as well as pharmacodynamic properties [24]. Such potential confounding factors may need to be controlled to detect reductions of drug susceptibility over time. Artesunate is known to rapidly metabolize in to its active metabolite, dihydroartemisinin that intern rapidly get absorb in the blood stream and result in to a rapid parasite clearance. The rate of elimination of artesunate is also rapid, with a half-life ranging from 2 to 3 h, and the partner drug pyronaridine is slow-acting drug with a longer half-life (13–17 days) [26]. Thus, the presence of parasites on Day 3 in this study might be due to the immune status of the patients, or parasite susceptibility to anti-malarial drugs and/or partner drug efficacy, which lags parasite clearance. The baseline median body temperature in the current study was 38.3 ± 0.94 °C and 78/90 (86.7%) had fever during enrollment. Fever clearance was rapid and a 100% clearance rate was observed on Day 3. A rapid fever clearance rate for pyronaridine–artesunate was reported from an efficacy study conducted in central and southern Vietnam [25]. In addition to the delay in parasite clearance, the dalliance in the clearance of fever might be suggestive of artemisinin resistance in Southeast Asia.

The median haemoglobin level at baseline (14.2 ± 2.03) was significantly higher than on Day 14 (12.7 ± 1.75), Day 28 (13.4 ± 1.52) and Day 42 (13.5 ± 1.79). This is not in line with previous studies on ACT where, following parasite clearance, haemoglobin levels had recovered. Several studies on other artemisinin-based combinations, such as artemisinin–lumefantrine, showed variable but consistent increase in haemoglobin level after treatment [27–29]. However, anaemia was reported as a severe adverse event in another study [30]. Decreased haemoglobin level after drug administration was found from other studies [31–33]. This might be partially explained by the fact that a transient and clinically moderate but significant decrease in haemoglobin after the treatment initiation with artemisinin derivative occurs due to haemolysis of parasitized and non-parasitized red cells [34].

The slight increase in median haemoglobin from day 14 to day 42 in this study is also consistent with another study [35]. It suggests that oral artemisinin derivative therapy may not have a late clinically relevant negative effect on haemoglobin. According to a preclinical study, artemisinin derivatives have been shown to induce reticulocytopaenia due to the suppression of erythroblasts [35], although the reticulocytes have not been described in this study. Haemolysis after treatment is reported to be associated with the treatment of severe malaria and hyperparasitaemic patients [31, 36]. In the current study comparison of median blood haemoglobin data among Day 14, Day 28 and Day 42 levels between patients with parasite density ≥ 10,000/µl and < 10,000/µl showed no significant difference.

Rehman et al. [36] reported delayed haemolysis 1–3 weeks after treatment with artemisinin derivatives. Artemisinin-based therapies cause rapid clearance of blood-stage parasite from the blood, with dead parasites cleared from erythrocytes by the spleen [36]. However, cleaned erythrocytes returned to the circulation generally have a reduced lifespan of about 7–15 days [37]. The shorter lifespan of infected erythrocytes may explain the timing of haemoglobin reduction after treatment. Haemolysis after treatment is an important adverse effect of artemisinin-based therapies [36].

Several adverse events were reported in this study. These events were similar to the symptoms of malaria, and there were no major adverse events observed. The most common adverse events observed were consistent with previous study. These studies have also reported that the adverse event profile for pyronaridine artesunate was similar to that of artemether–lumefantrine and mefloquine–artesunate in falciparum malaria [38–40]. Although headache, cough and mouth ulcer are common symptoms of malaria, their persistence after the recovery of other malaria symptoms observed in this study makes them potential drug-related adverse events. But more study may be necessary to classify the events as drug-related or due to other circumstances.

### Limitation of the study

The study has several limitations: blood drug level was not measured, advanced methods such as the molecular detection of standard resistance markers are yet to be done. The potential reduction of haemoglobin level after pyronaridine artesunate treatment and transaminase level were not investigated. The study enrolled only on adults (excluding vulnerable children), and careful consideration may be required in generalizing the study outcomes.

## Conclusions

In the first therapeutic efficacy study in Ethiopia, pyronaridine–artesunate was highly efficacious for the treatment of uncomplicated *P. falciparum* malaria in adults, with rapid parasite clearance and fever resolution. Serious drug adverse events were not observed, implying that the drug is safe for the study group. (Additional file 1).

## Supplementary information

**Additional file 1: Annex 1. **Kaplan-Meier Analysis without PCR correction.

## Data Availability

The datasets used and/or analysed during the current study are available from the corresponding author and EPHI on a reasonable request.

## Abbreviations

ACT: Artemisinin-based combination therapy
APCR: Adequate parasitological and clinical response
DBS: Dry blood spot
DHA: Dihydroartemisinin
EPHI: Ethiopian Public Health
ETF: Early treatment failure
FMoH: Federal Ministry of Health
Hb: Haemoglobin
IQR: Interquartile Range
K-M: Kaplan Meier
PCR: Polymerase chain reaction
LFU: Lose of follow up
OPD: Outpatient department
PP: Per protocol
RBC: Red blood cell
TF: Treatment failure
WBC: White blood cell
WHO: World Health Organization
