# Supplementary material for: Therapeutic efficacy of pyronaridine-artesunate (Pyramax®) against uncomplicated Plasmodium falciparum infection at Hamusit Health Centre, Northwest Ethiopia
Source: Malar J. 2023 Jun 17;22:186. doi: 10.1186/s12936-023-04618-y (PMC10276904; doi:10.1186/s12936-023-04618-y)
Supplement: Supplementary file 1 — Additional file 1: Annex 1. Kaplan-Meier Analysis without PCR correction. [file 12936_2023_4618_MOESM1_ESM.docx]

**Annex 1**

Kaplan-Meier Analysis without PCR correction

(A) Without PCR correction
